# Ancestry dependent balancing selection of placental dysferlin at high-altitude

**DOI:** 10.3389/fcell.2023.1125972

**Published:** 2023-03-21

**Authors:** William E. Gundling, Sasha Post, Nicholas P. Illsley, Lourdes Echalar, Stacy Zamudio, Derek E. Wildman

**Affiliations:** ^1^ Department of Biology, Christian Brothers University, Memphis, TN, United States; ^2^ College of Public Health, University of South Florida, Tampa, FL, United States; ^3^ Placental Research Group LLC., Maplewood, NJ, United States; ^4^ Instituto Boliviano de Biología de Altura, Universidad de San Andreas Mayor, La Paz, Bolivia

**Keywords:** placenta, cell fusion, dysferlin, methylation, genomics

## Abstract

**Introduction:** The placenta mediates fetal growth by regulating gas and nutrient exchange between the mother and the fetus. The cell type in the placenta where this nutrient exchange occurs is called the syncytiotrophoblast, which is the barrier between the fetal and maternal blood. Residence at high-altitude is strongly associated with reduced 3rd trimester fetal growth and increased rates of complications such as preeclampsia. We asked whether altitude and/or ancestry-related placental gene expression contributes to differential fetal growth under high-altitude conditions, as native populations have greater fetal growth than migrants to high-altitude.

**Methods:** We have previously shown that methylation differences largely accounted for altitude-associated differences in placental gene expression that favor improved fetal growth among high-altitude natives. We tested for differences in DNA methylation between Andean and European placental samples from Bolivia [La Paz (∼3,600 m) and Santa Cruz, Bolivia (∼400 m)]. One group of genes showing significant altitude-related differences are those involved in cell fusion and membrane repair in the syncytiotrophoblast. Dysferlin (*DYSF*) shows greater expression levels in high- vs. low-altitude placentas, regardless of ancestry. *DYSF* has a single nucleotide variant (rs10166384;G/A) located at a methylation site that can potentially stimulate or repress *DYSF* expression. Following up with individual DNA genotyping in an expanded sample size, we observed three classes of DNA methylation that corresponded to individual genotypes of rs10166384 (A/A < A/G < G/G). We tested whether these genotypes are under Darwinian selection pressure by sequencing a ∼2.5 kb fragment including the *DYSF* variants from 96 Bolivian samples and compared them to data from the 1000 genomes project.

**Results:** We found that balancing selection (Tajima’s D = 2.37) was acting on this fragment among Andeans regardless of altitude, and in Europeans at high-altitude (Tajima’s D = 1.85).

**Discussion:** This supports that balancing selection acting on dysferlin is capable of altering DNA methylation patterns based on environmental exposure to high-altitude hypoxia. This finding is analogous to balancing selection seen frequency-dependent selection, implying both alleles are advantageous in different ways depending on environmental circumstances. Preservation of the adenine (A) and guanine (G) alleles may therefore aid both Andeans and Europeans in an altitude dependent fashion.

## Introduction

Natural selection is an evolutionary mechanism contributing to differences between populations at both the genotypic and phenotypic levels (Barreiro et al., 2008). Two major types of natural selection cause changes in gene allele frequencies; directional selection and balancing selection. A locus under directional selection in a population has one allele frequency that is significantly greater than the other suggesting the more frequent allele is beneficial compared to the less frequent allele (Smith and Haigh, 1974). An example of positive directional selection in humans is lactase persistence among individuals residing in arid regions (Tishkoff et al., 2007). Three major sequence variants within the lactase gene show positive selection. Functionally, these variants promote better absorption of nutrients and water from milk, providing an adaptive advantage in arid regions. Conversely, a locus experiencing balancing selection has two alleles of roughly equal frequency suggesting both alleles may be beneficial (Dobzhansky and Dobzhansky, 1970). A well-known example of balancing selection is seen in those individuals who are heterozygotes for the sickle cell allele associated with hemoglobin beta (Allison, 1954; Hoff et al., 2001). Heterozygous individuals have mostly healthy erythrocytes (i.e., RBC) but also have resistance to malaria infection of these same erythrocytes (Allison, 1954). In contrast, homozygous individuals either are more vulnerable to malaria infection or have sickled erythrocytes that impair oxygen delivery. Therefore, the heterozygote condition is favored by natural selection as it enables efficient transport of oxygen while simultaneously preventing malaria infection. In contrast, homozygotes are disadvantaged depending on environmental context. In evolutionary theory, this phenomenon is called heterozygote advantage. Balancing selection also occurs through frequency-dependent selection, where two alleles have intermediate frequencies in which both alleles have roughly equal allele frequencies, known as balanced polymorphisms suggesting that both alleles may be beneficial (Hedrick, 2007). Examples of frequency dependent selection seen in humans include allelic variation in the human leukocyte antigens where an intermediate number of alleles may allow for greater recognition of pathogens (Brandt et al., 2018). In contrast to selection pressures, there are also random events that change allele frequencies. Genetic drift, for example, is often found in small populations isolated by migration, geography and/or culture. Drift is caused by both founder effects, where a small founder population is not genetically representative of the parent population (Tournebize et al., 2022) and population bottlenecks (rapid loss of population resulting in a different genetic make-up of the remaining individuals) (Amos and Hoffman, 2010; Masel, 2011). We can distinguish between positive selection, balancing selection, and genetic drift by calculating the Tajima’s D value for a DNA sequence (Tajima, 1989).

The high-altitude environment exerts selection pressure, resulting in allele frequency changes in different regions of the human genome. Early studies among Tibetans (Beall et al., 2010) and Andeans (Bigham et al., 2013) found signatures of positive selection in genes associated with the hypoxia-inducible factors (HIFs), transcription factors critical to oxygen homeostasis. These include genes encoding endothelial PAS domain protein 1 (HIF-2 alpha) and Egl-9 family hypoxia inducible factor 1 (PHD2, a prolyl hydroxylase involved in HIF regulation).

**FIGURE 1:**
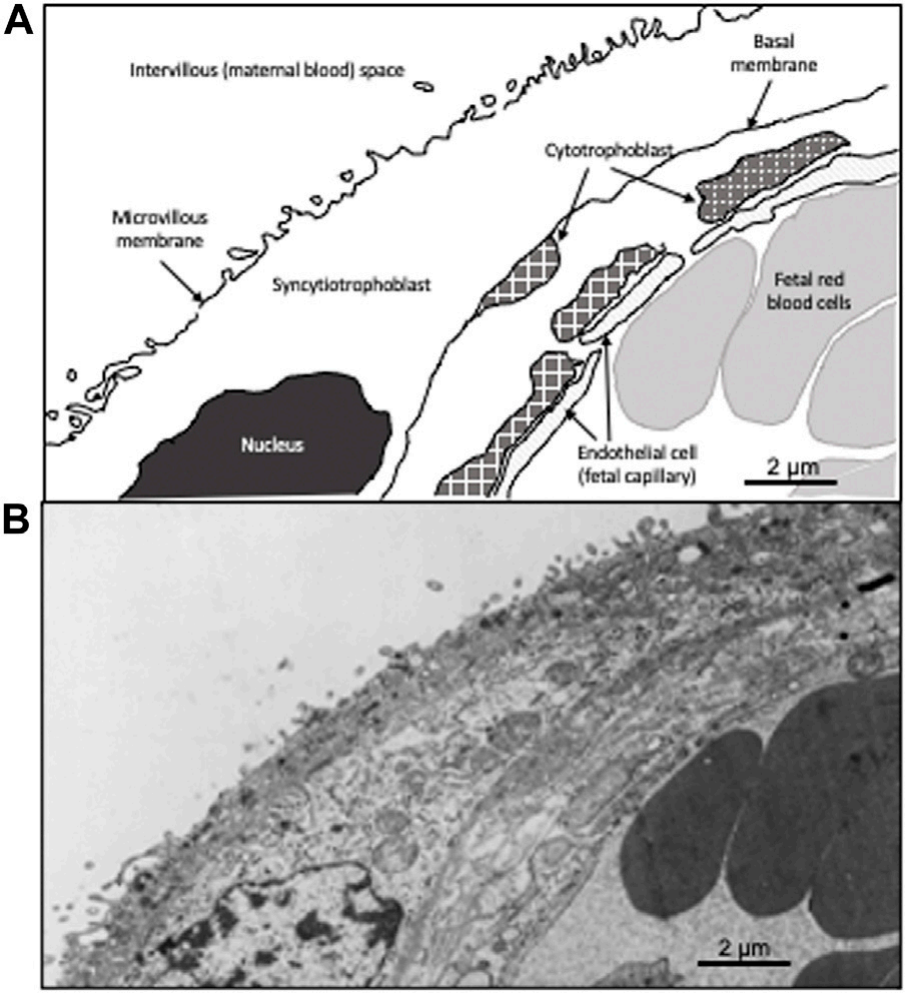
Drawing of a villous cross-section of a human placenta. A cross-section of the villous tissue of the human placenta. **(A)** is a schematic showing the composition the maternal fetal interface. The outer layer is comprised of multi-nucleated syncytiotrophoblast in contact with the maternal blood. The second layer is comprised of mononucleated cytotrophoblast, which fuse to form the syncytiotrophoblast. Below the cytotrophoblast is the endothelial wall of the fetal capillary. Fetal red blood cells (erythrocytes) are present in the fetal capillary. **(B)** shows the transmission electron micrograph of the placenta that was used to generate **(A)**. The dark cells on the right are fetal erythrocytes while the space on the upper left is the maternal blood (intervillous) space. The two circulatory systems are separated by the syncytiotrophoblast and the cytotrophoblast.

We found that placental genes associated with cell fusion and proliferation, critical processes in placental development, were differentially expressed between individuals residing at high and low-altitude. Of 36 altitude-associated differentially expressed genes, 8 had significant correlation between gene expression and DNA methylation (Gundling et al., 2018). One of these is dysferlin (*DYSF*). Dysferlin is linked to the development of syncytial cells and membrane repair. *DYSF* is a gene spanning roughly 230 Kb with more than 50 coding exons. Within the third intron of *DYSF,* there is a single nucleotide polymorphism (SNP) that causes that site to either be a Guanine (G) or and Adenine (A) that changes a CpG site to a CpA. CpG sites are commonly methylated, whereas the change from a G to A makes methylation less likely as methylation rarely occurs at CpA sites (Patil et al., 2014). Methylation is a common epigenetic mark that can be induced not only in developmental processes as a regulated event, but also by environmental change (Uddin et al., 2010). *DYSF* encodes a membrane bound protein associated with calcium regulated membrane repair (Codding et al., 2016). *DYSF* plays an important role in the formation of syncytial tissues, which contain multinucleated cells formed by the fusion of mononucleated cells (De Luna et al., 2004; Han and Campbell, 2007; Omata et al., 2013) (Figure 1). In the placenta, dysferlin is expressed in the apical membrane (facing the maternal blood) of the multi-nucleated syncytiotrophoblast. Expression is induced when the mononucleated cytotrophoblast fuse during syncytiotrophoblast formation and repair (Vandre et al., 2007). Methylation likely plays a role in regulating gene expression of *DYSF*. It is known to be a methylation driven gene in other cells such as monocytes in cardiovascular diseases (Zhang et al., 2022).

We sought to test whether natural selection, induced by the high-altitude environment, acted on dysferlin ultimately changing allele frequency in relation to DNA methylation and gene expression. We examined whether the population was in Hardy-Weinberg equilibrium and distinguished between selection and genetic drift using statistical tests (i.e., Tajima’s D). We found that individuals residing at high-altitude had a signature of frequency-dependent balancing selection, suggesting that the possession of both alleles (A and G) may be beneficial for Andean and European individuals at high-altitude. We have previously posited that Andean individuals have genetically adapted to their high-altitude environment by methylation-favored, and therefore environmentally modifiable placental physiology (Gundling et al., 2018). In the case of dysferlin, we hypothesized that the heterozygote dysferlin alleles would be more prevalent among Andeans, whereas their European counterparts (i.e., those of Spanish descent) would not yet have evolved allele frequencies reflecting balancing selection. We tested this proposition by comparing allele frequencies in high- and low-altitude Bolivian populations of both indigenous and European descent.

## Materials and methods

### Samples

Term placentas from 96 Bolivian individuals were collected from La Paz, Bolivia (high-altitude; 3,600 m *n* = 48) and Santa Cruz, Bolivia (low-altitude; 400 m *n* = 48). Of our 96 individuals 48 were of European ancestry and 48 were of Andean ancestry. The placentas were from healthy, term (≥ 37 weeks gestation), singleton pregnancies in individuals without pregnancy complications delivered *via* elective caesarian section. All women gave written informed consent to protocols approved by the Bolivian National Bioethics Committee, the Universidad Mayor de San Andrés, Instituto Boliviano de Biología de Altura Consejo Tecnico, and the United States Institutional IRB from the University of Medicine and Dentistry, New Jersey Medical School in Newark, NJ (now Rutgers). Tissue for transmission electron microscopy was rinsed three times over a 24-h period in 0.1 M sodium cacodylate, pH 7.4, and then post-fixed in 1% osmium tetroxide in cacodylate buffer at 4°C for 1 h. After a brief rinse in buffer the tissue was dehydrated in ascending concentrations of alcohol, embedded in Taab resin, and polymerized at 60°C for 36–48 h. Ultrathin sections were cut on an LKB ultramicrotome, and grids were stained with uranyl acetate and Reynold’s lead citrate prior to examination in a Phillips EM 301 electron microscope (Figure 1).

### Identification of differentially methylated sites associated with differential gene expression

DNA was extracted from the villous tissue of term placenta and used for DNA methylation analysis and SNP genotyping. DNA methylation data was generated using the HM450k methylation microarray chips using previously described methods (Gundling et al., 2018). Validation was performed using 350 ng of placental DNA from 8 individuals from our sample groups as well as high and low methylation controls (Zymo Research, Irvine, CA). Samples were bisulfite converted using the Zymo EZ DNA Methylation-Gold Kit (Zymo Research, Irvine, CA). The samples chosen for pyrosequencing were those that had methylation microarray data used in Gundling et al. (2018), and provided an even representation of each dysferlin genotype (G/G, G/A, or A/A). Primers developed for this study were designed using the PyroMark Q24 Assay Design Software 2.0 (Qiagen, Hilden, Germany) (Supplementary Table S1). The amplicon fragment was ∼60 bp containing three different CpG sites and was amplified using the Qiagen PyroMark PCR master mix and the manufacturer's suggested protocol (Supplementary Table S1). Pyrosequencing was performed on the PyroMarkQ24 Advanced using a sequencing primer (Supplementary Table S1) as well as the standard PyroMarkQ24 Advanced reagents and protocol.

### Single nucleotide polymorphism genotyping

We genotyped SNP rs10166384 within dysferlin (*DYSF*) using DNA from term placentas of 96 individuals (48 Andean, 48 European) collected from La Paz, Bolivia (3,600 m) and Santa Cruz, Bolivia (400 m). These included the 45 individuals from our prior study in which three methylation patterns for DYSF were observed (Gundling et al., 2018). We genotyped SNP rs10166384 in all 96 individuals using a TaqMan SNP genotyping assay (TaqMan assay C__387100_10). This probe was 50 nucleotides long with rs10166384 (A/G) being located at the 26th base. The Adenine was labeled with VIC and the Guanine was labeled with FAM. The assay was run on an ABI 7500 qPCR machine (Applied Biosystems, CA). Standard run conditions used were used by the TaqMan SNP genotyping assay provided in the ABI 7500 manual. We then calculated the allele frequencies of the A and G allele for each of the four sample populations. We then tested to see if the populations are in Hardy-Weinberg equilibrium. When a population is within Hardy-Weinberg equilibrium it is assumed to have no selection, no mutation, no migration, a large population size, and random mating. Chi-square analysis was performed to determine if the proportion of genotypes in each of the four sample groups were significantly outside of Hardy-Weinberg equilibrium (*p* < 0.05).

### Detecting natural selection

To determine if natural selection was acting on the SNP of interest (rs10166384) we amplified a ∼2,500 base pair region using polymerase chain reaction from the placental DNA of the 96 pregnancies (Supplementary Table S1). PCR products were purified using the QIAquick PCR purification kit (Qiagen, Hilden, Germany). Between 30 and 50 μg of each PCR product was sequenced at the University of Illinois, Urbana Champaign (UIUC) core sequencing center (Urbana, IL). The sequences generated from each primer were aligned using Sequencher (Gene Codes, Ann Arbor, MI). The complete sequences from each sample were then aligned to each other using the ClustalW (Thompson et al., 1994) algorithm implemented in MEGA version 6.06 (Tamura et al., 2013). Once sequences were aligned, a variant call format file (vcf file) was generated using SNP-sites (Page et al., 2016). We calculated allele frequencies for each polymorphism, determined if each subpopulation was in Hardy-Weinberg equilibrium, and calculated Tajima’s D for all SNPs with a minor allele > 0.05 using vcftools version 0.1.13 (Tajima, 1989; Danecek et al., 2011).

## Results

**FIGURE 2:**
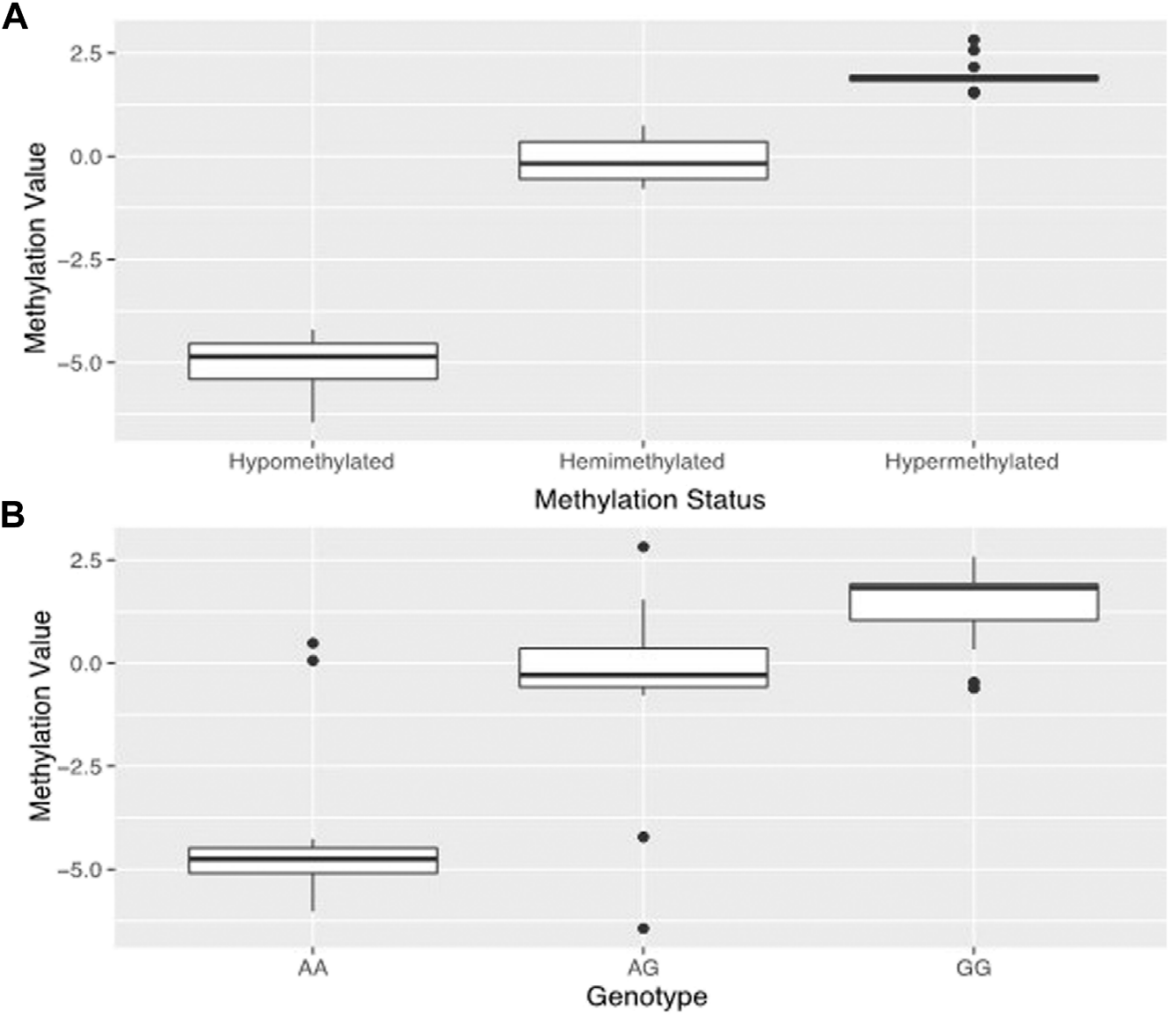
Estimated genotypes based on methylation. Prediction and confirmation of genotype using DNA methylation status at methylation probe cg09829645 which is in the same location as dysferlin variant rs10166384. Data used to generate **(A)** was taken from the 45 samples used in the original study. **(A)** shows the tripartite distribution of methylation levels detected for dysferlin at HM450k methylation probecg09829645 in the 45 placentas from our original study (Gundling et al., 2018). One group is hypomethylated, one group is hemimethylated, and one group is hypermethylated. **(B)** shows the mean methylation estimates for genotypes sampled at the rs10166384 variant in 96 placentas including the 45 from the 2018 study. Those with the AA genotype have low levels of methylation, those with GG genotype have high levels of DNA methylation and those with the AG genotype tend to have methylation levels that fall in between the two homozygous groups. Methylation values (M-values) were calculated according to Du et al., 2010.

By analyzing the DNA methylation patterns of genes we previously found to be differentially expressed (Gundling et al., 2018), we found a probe (cg09829645) within the third intron of dysferlin that had three distinct levels of DNA methylation (Figure 2A).

One group showed hypomethylation with low levels of DNA methylation (M = −5.01 ± 0.67), one with hypermethylated, high levels of DNA methylation (M = 1.97 ± 0.36), and one hemi-methylated with median DNA methylation levels (M = −0.11 ± 0.49). This probe is in the same position as the polymorphism rs10166384 and can be either a Guanine (G) or an Adenine (A) nucleotide. By genotyping this site in the 96 individuals of Andean and European descent from high and low-altitude, we found that the levels of DNA methylation corresponded to the three possible different genotypes at rs10166384 (Figure 2B). Those that were homozygous for the A allele (A/A) had on average the lowest levels of DNA methylation (M = −4.21 ± 1.96), those that there homozygous for the G allele (G/G) had on average the highest levels of DNA methylation (M = 1.41 ± 0.97), and those that were heterozygous (A/G) had methylation values that fell in between the two homozygous groups (M = −0.51 ± 2.06) (Figure 2B). The genotype-specific DNA methylation was confirmed through pyrosequencing a region containing rs10166384 in a subset of individuals for which we have methylation microarray data for probe cg09829645 (Supplementary Figure S1) (Gundling et al., 2018).

Individuals residing at high-altitude were more likely to have the A allele (Frequency of A = 0.59) and those residing at low-altitude were more likely to have the G allele (Frequency of G = 0.58, Table 1). The allele frequencies varied depending on ancestry as well. Europeans residing at high-altitude had higher A allele frequency than Europeans residing at low-altitude and a greater number of individuals that were homozygous for the A allele (Table 1). Those of primarily European descent had a negative correlation between the A allele frequency and Native American admixture, previously estimated in Zamudio et al. (2007). Europeans residing at high-altitude had 26% indigenous Bolivian admixture and an A allele frequency of 0.64 while low-altitude Europeans had greater indigenous admixture (33%) and the frequency of the A allele was 0.38 (Table 1). Among Andeans there was a less drastic change between high and low-altitude Andeans with A allele frequences of 0.54 and 0.46 respectively. There was also less admixture among the Andean populations compared to the European populations. The Andeans residing at high-altitude had ∼3% European admixture and those Andeans residing at low-altitude had ∼5% European admixture.

**TABLE 1:** Allele frequency and genotype proportions for site rs10166384 among the Bolivian samples as well as East Asian and European populations from the 1,000 genomes project.

Population	A frequency	G frequency	AA frequency	AG frequency	GG frequency
CHB (*n* = 206)	0.28	0.72	0.08	0.60	0.32
LE (*n* = 24)	0.38	0.62	0.10	0.36	0.54
L (*n* = 48)	0.42	0.58	0.15	0.54	0.31
LA (*n* = 24)	0.46	0.54	0.22	0.48	0.30
A (*n* = 48)	0.50	0.50	0.28	0.45	0.27
E (*n* = 48)	0.50	0.50	0.32	0.36	0.32
HA (*n* = 24)	0.54	0.46	0.42	0.25	0.33
H (*n* = 48)	0.59	0.41	0.46	0.26	0.28
HE (*n* = 24)	0.64	0.36	0.49	0.39	0.12
IBS (*n* = 214)	0.68	0.32	0.50	0.27	0.23

Allele and genotype frequencies site rs10166384 for the Bolivian sample populations and representative East Asian (CHB) and European (IBS) populations, LE, low-altitude Europeans; L, low-altitude, LA, low-altitude Andeans; A, Andeans; E, Europeans; HA, high-altitude Andeans, H, high-altitude; HE, high-altitude Europeans; IBS, Iberian Spanish.

We tested to see if natural selection induced by the high-altitude environment was contributing to the change in allele frequency at the location of SNP rs10166384, which is associated with the shift in DNA methylation. Hardy-Weinberg equilibrium was present in both Andeans and Europeans residing at low-altitude. Andeans residing at high-altitude were not in Hardy-Weinberg equilibrium (χ^2^ = 5.916, *p* = 0.015), indicating the population is being influenced by selection pressure with respect to these alleles. High-altitude Andeans had fewer heterozygotes than a population in accordance with Hardy-Weinberg equilibrium (Table 1). For high-altitude Europeans being out of Hardy Weinberg equilibrium was of borderline significance (χ^2^ = 3.711, *p* = 0.054). European high-altitude residents, like Andeans had fewer heterozygotes than would be expected for a population in Hardy-Weinberg equilibrium.

We then tested whether the change in genotype proportions was influenced by directional selection, balancing selection, or random genetic drift by calculating Tajima’s on a 2.5 kb region within the third intron of *DYSF* containing six polymorphisms with minor allele frequencies > 0.05 (Tajima, 1989). We found that the Andeans, regardless of altitude, had the highest estimated Tajima’s D (Tajima’s D = 2.35) suggesting balancing selection on roughly equal frequencies of each allele. While lower than in Andeans, Europeans, regardless of altitude, also had a Tajima’s D suggesting balancing selection (Tajima’s D = 1.86).

## Discussion

### Nucleotide adaptation in dysferlin

The dysferlin single nucleotide variant rs10166384 (G/A) is located directly downstream of a cytosine causing a CpG site to potentially become a CpA site. This variation reduces the likelihood of DNA methylation (McClay et al., 2015). rs10166384 is located within a *TEAD4* transcription factor binding site as seen using the UCSC genomes browser, involved in the formation of syncytial tissues This included the syncytiotrophoblast, the outer layer of the placental villi (Figure 1) (Benhaddou et al., 2012) facing the maternal intervillous blood that provides the oxygen and nutrients required by the placenta and fetus. Increased DNA methylation along the TEAD4 transcription factor binding sites limits binding of the TEAD4 transcription factor, resulting in decreased *DYSF* gene expression (Gornikiewicz et al., 2016) Recent studies indicate *DYSF* expression is driven by DNA methylation in other cell types as well (Zhang et al., 2022). We would therefore expect that *DYSF* AA homozygotes would have more, GG homozygotes less, and AG heterozygotes intermediate levels of dysferlin protein in the syncytiotrophoblast.

There are only 3 true syncytial tissues in humans: muscle fibers, osteoclasts or giant cells and the placental syncytiotrophoblast (the latter is shown in Figure 1) (Benhaddou et al., 2012). It is unsurprising, given dysferlin’s role in the muscle sarcolemma, that numerous pathogenic mutations in the dysferlin gene are linked to muscle wasting diseases known collectively as dysferlinopathies and attributable to failure of muscle repair mechanisms (Charnay et al., 2021). In the human placenta, dysferlin localizes strictly to the syncytiotrophoblast (Robinson et al., 2009) DYSF plays a key role in syncytiotrophoblast fusion with the underlying mononuclear cytotrophoblast (Omata et al., 2013) (Robinson et al., 2009). Cytotrophoblasts are progressively incorporated into the syncytium *via* fusion over the course of pregnancy. This critically important process allows the syncytium to expand as the placenta grows. From this perspective an increase in dysferlin at high-altitude might be beneficial. This is supported by placental morphological studies indicating reduced perivillous fibrin deposition is a prominent difference between high- and low-altitude placentae (Zamudio, 2003) Perivillous fibrin deposition is related to syncytial damage and breaks, occasionally pathological, and associated with the placental hypoxia seen in preeclampsia IUGR (Lang et al., 2009). Additional support for the idea that elevated DYSF would be beneficial at high-altitude derives from the well-supported evidence that hypoxia, including that due to high-altitude, causes ballooning of the tertiary villi, expanding and thinning the syncytium due to capillary growth, reducing syncytiotrophoblast thickness and permitting greater oxygen diffusion (Burton et al., 1996; Mayhew, 2003). Nonetheless, other studies suggest altitude decreases trophoblast growth (Mayhew et al., 2002), while molecular studies indicate the mechanisms regulating trophoblast turnover into the syncytium are decreased (Soleymanlou et al., 2007).

The population sub-group with the highest A allele frequencies are the high-altitude Europeans (A allele frequency = 0.64). The higher A allele frequency corresponds with a decrease in methylation compared to low-altitude Europeans (ΔM = −2.62). Lower DNA methylation may contribute to the higher *DYSF* gene expression within the high-altitude European population compared to the low-altitude European population. The A allele is also more abundant among those individuals residing in Spain who were genotyped as part of the 1,000 genomes project, suggesting that the A allele is the ancestral European allele. The high frequency of A alleles among Europeans residing at high-altitude, which differed significantly from that of low-altitude Europeans (0.64 at 3,600 m vs. 0.38 at 400 m) could be related to the fact that we only examined individuals with healthy pregnancies. While no single factor can be said to influence placental health, the large difference in gene frequency suggests the A allele may be under greater selection pressure among migrants than natives to high-altitude because it may be linked to more effective *DYSF* transcription and therefore better cytotrophoblast fusion and syncytiotrophoblast repair. We predict that if we were to genotype Europeans residing at high-altitude who experienced problematic pregnancies, we would see increased G allele frequencies.

**FIGURE 3:**
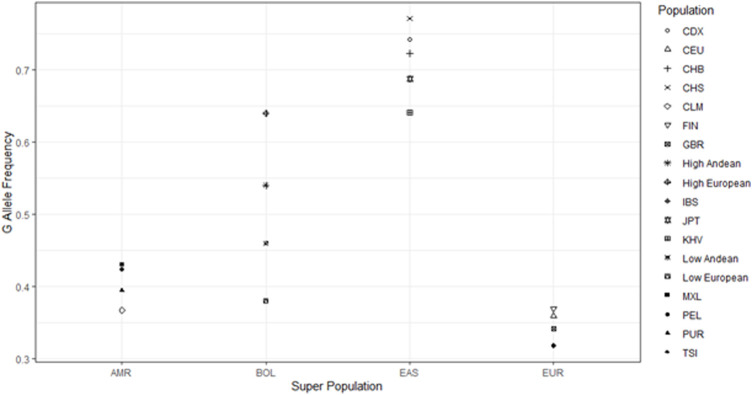
Comparisons of Andean allele frequency to 1,000 genomes data. Figure shows the comparison of the G allele frequency among our Andean sample populations in relation to the 1,000 genomes populations. AMR = Americans from the 1,000 genomes project, BOL = Samples from Bolivia residing at both high and low-altitude, EAS = East Asians from the 1,000 genomes project, EUR = Europeans from the 1,000 genomes project. The Populations include groups from the 1,000 genomes project (CDX = Chinese Dai, CEU = European from Utah, United States, CHB = Han Chinese from Beijing, CHS = Han Chinese from South China, CLM = Colombian, FIN = Finnish, GBR = British, IBS = Ibererian from Spain, JPT = Japanese, KHV = Vietnamese, MXL = Mexican from Los Angeles, PEL = Peruvian, PUR = Puerto Rican, TSI = Italian). We also included our 4 sample populations from Bolivia (High Andean = High altitude Andean, High European = High altitude European, Low Andean = Low altitude Andean, Low European = Low altitude European).

We compared allele frequencies of our sample populations to those of East Asian, European, and South American descent from the 1000 genomes project (1000 Genomes Project Consortium et al., 2015). The allele frequencies of our Bolivian samples lie in between those of the East Asian and European 1000 genomes populations (Figure 3). We chose to look at the east Asian populations because, while the founding population for North and South American paleo-Indian migrations is lost, they likely contain the descendants of the founding population for Native Americans, which crossed over the Bering Land Bridge roughly 15,000–17,000 years ago (Perego et al., 2010). The East Asian participants that were part of the 1,000 genomes project had G as the major allele for SNP rs10166384 with an average G allele frequency of 0.74, whilst Andeans had frequencies of 0.46 and 0.54 at low and high-altitude respectively. Apart from the East Asians, the European samples from the 1,000 genomes had the A as the major allele at this site with an A allele frequency of 0.68, close to that of high-altitude Europeans. The fact that both the Andeans and Europeans residing in Bolivia (regardless of altitude) had roughly equal frequencies of both the A and G allele suggests that there has been East Asian and European admixture resulting in the Andeans gaining more of the European A allele and the Europeans gaining more of the East Asian G allele (Figure 3). This implies that the more generations Europeans have resided at high-altitude the greater the chance that they will inherit native admixture. This would result in an increase in the frequency of G allele causing them to become significantly out of Hardy-Weinberg equilibrium.

We found that the Native Americans residing at high-altitude had genotype proportions that were significantly out of Hardy-Weinberg equilibrium (*p* = 0.015). This suggests that over the millennia the Andeans have resided at high-altitude the selection pressure of the environment has caused a shift in genotype proportions. This shift, exemplified by rs10166384 is an example of how evolution may favor DNA methylation to alter not just *DYSF*, but other gene expression as populations shifts between high and low-altitude environments (Gundling et al., 2018). Likewise, Europeans residing at high-altitude are of borderline significance for being out of Hardy-Weinberg equilibrium (*p* = 0.054). This indicates that the selection pressure of high-altitude may also be resulting in sequence level change at site rs10166384. However, the Europeans have not resided at high-altitude for enough generations to cause the population to be significantly out of Hardy-Weinberg equilibrium. We speculate that after more generations at high-altitude, or even with a larger sample size of European ancestry high-altitude residents rs10166384 will be out of Hardy-Weinberg equilibrium.

That our Bolivian Andean and European populations regardless of altitude had A and G allele frequencies of ∼0.5 supports that balancing selection is acting on and around variant rs10166384. This is supported by the positive Tajima’s D value of 2.37 and 1.85 across Andean and European populations respectively. A significantly positive Tajima’s D (D > 1.80) indicates balancing selection suggesting that both the A and G allele maybe beneficial in the high-altitude environment (Tajima, 1989). Those possessing a G allele are more likely to be methylated limiting the binding of the TEAD4 transcription factor resulting in decreased *DYSF* gene expression. This is supported by the findings in our prior study where were found increased gene expression of *DYSF* at high altitude suggesting that the increase frequency of the A allele at high-altitude is increasing gene expression by limiting DNA methylation (Gundling et al., 2018). Methylation at this site may allow for more transient regulation of dysferlin allowing for a smoother transition between high and low-altitude. Those that have the A allele may be regulating gene expression by limiting the ability of this CpG site to be methylated potentially resulting in higher gene expression. The intermediate allele frequencies suggest that balancing selection is acting in a frequency dependent manner rather than through heterozygous advantage (Brandt et al., 2018). While genotypes cannot be induced, methylation status of particular genotypes may potentially be inducible based on environmental conditions. The preservation of both the A and G alleles among native Andeans regardless of altitude suggests that both the ability to be methylated and consequent impacts on transcription factor binding may aid the Andeans in attaining greater fetal growth and better placental health regardless of the altitude they reside in at the time.

One of the major limitations of our study is the small sample size for our Bolivian population, although we did increase our sample size from 45 to 96 samples to help combat this issue. Increasing our sample size further may help increase our statistical power to detect meaningful differences among groups. Another limitation is that we focused only on one site within one gene to test for balancing selection. Future studies should involve increased number of sites associated with altitude dependent differentially expressed genes to detect whether selection occurred at sites impacted by DNA methylation.

## Conclusion

We identified an environmentally challenged population (high-altitude) in which ancestry dependent natural selection may induce altered DNA methylation status, thereby benefitting placental function. In the gene dysferlin, we found a variant (rs10166384) located within a transcription factor-binding site that can alter dysferlin gene expression. Those individuals with the A allele who reside at high-altitude are likely to have decreased DNA methylation and higher dysferlin gene expression. The higher dysferlin gene expression is likely to contribute to more effective syncytiotrophoblast turnover and repair in response to the high-altitude environment, which may lead to more successful pregnancies at high-altitude. Those with the G allele are more likely to have DNA methylation along the transcription factor-binding site and decreased gene expression. This may be advantageous among those of Andean ancestry because it may allow them a way to easily modify gene expression of those genes regulated by the TEAD4 transcription factor as they move between high and low-altitude. As native Andeans move to high-altitude the DNA methylation may be removed increasing dysterlin gene expression and allowing for increased membrane repair compared to low-altitude.

## Data Availability

The datasets presented in this study can be found in online repositories. The names of the repository/repositories and accession number(s) can be found below: https://www.ncbi.nlm.nih.gov/geo/, GSE100988 data released by NIH on publication.

## Supplementary material

SUPPLEMENTARY FIGURE S1 Correlation of 450 k methylation with pyrosequencing. Correlation of beta values generated using the Illumina 450 k methylation microarray (x-axis) and percent methylation calculated from the pyrosequencing analysis. The gray band surrounding the blue regression line represents the 95% confidence interval. We see that those samples with the AA genotype demonstrated minimal methylation (i.e., Beta values close to 0%) when analyzed through methylation array as well as pyrosequencing. Those with the heterozygous genotype (GA) had roughly (beta values representing 50% percent methylation). Lastly those that were homozygous for the G allele had the highest methylation values with Beta values indicating approximately 70% methylation.
