# Supplementary material for: Ancestry dependent balancing selection of placental dysferlin at high-altitude
Source: Front Cell Dev Biol. 2023 Mar 21;11:1125972. doi: 10.3389/fcell.2023.1125972 (PMC10070852; doi:10.3389/fcell.2023.1125972)
Supplement: Supplementary file 1 [file Table1.DOCX]

**Supplementary Table 1: Primers used for PCR, Sanger sequencing ,and pyrosequencing**

| Primer Name | Primer Sequence (5'-3') | Annealing Temperature (°C) | Amplicon length(bp) |
| --- | --- | --- | --- |
| DYSF_F1 | ATGCCATCCCTGCATAGTGT | 56.6 | 851 |
| DYSF_R1 | AGCCTCCTGTGCCATCTTAG |  |  |
| DYSF_F3 | CCTGCTGGTAAGTGAGGAGT | 56 | 2051 |
| DYSF_R4 | GGCCAGCTAACCATAGAAAA |  |  |
| DYSF_F5 | CCCCAAACCTCCATGATTTA | 53 | 675 |
| DYSF_R5 | CATAATCCCCATGTGCTGTG |  |  |
| DYSF_PyroF1 | GGTTTGGGGATTGGTTAGTT | 54 | 110 |
| DYSF_PyroR1-Biotin | Biotin-TCCCCTATCCTAAACACCATAAA |  |  |
| Sequencing Primer |  |  |  |
| DYSF_Pyro_S1 | GTTTGAATAATTTTTGTGGG |  |  |

**Supplementary Table 1:** Table of primers used for PCR, Sanger sequencing and pyrosequencing. Primers DYSF_F1, DYSF_R1, DYSF_F3, DYSF_R4, DYSF_F5, and DYSF_R5 were primer sets used for the detection of natural selection. Primers DYSF_PyroF1 and DYSF_PyroR1-Biotin were primers used on bisulfite converted DNA to generate the amplicon used for the vaiidation of the methylation array. Primer DYSF_Pyro_S1 was the sequencing primer used as part of the pyrosequencing protocol.
