# Supplementary figures and images for: Ancestry dependent balancing selection of placental dysferlin at high-altitude
Source: Front Cell Dev Biol. 2023 Mar 21;11:1125972. doi: 10.3389/fcell.2023.1125972 (PMC10070852; doi:10.3389/fcell.2023.1125972)

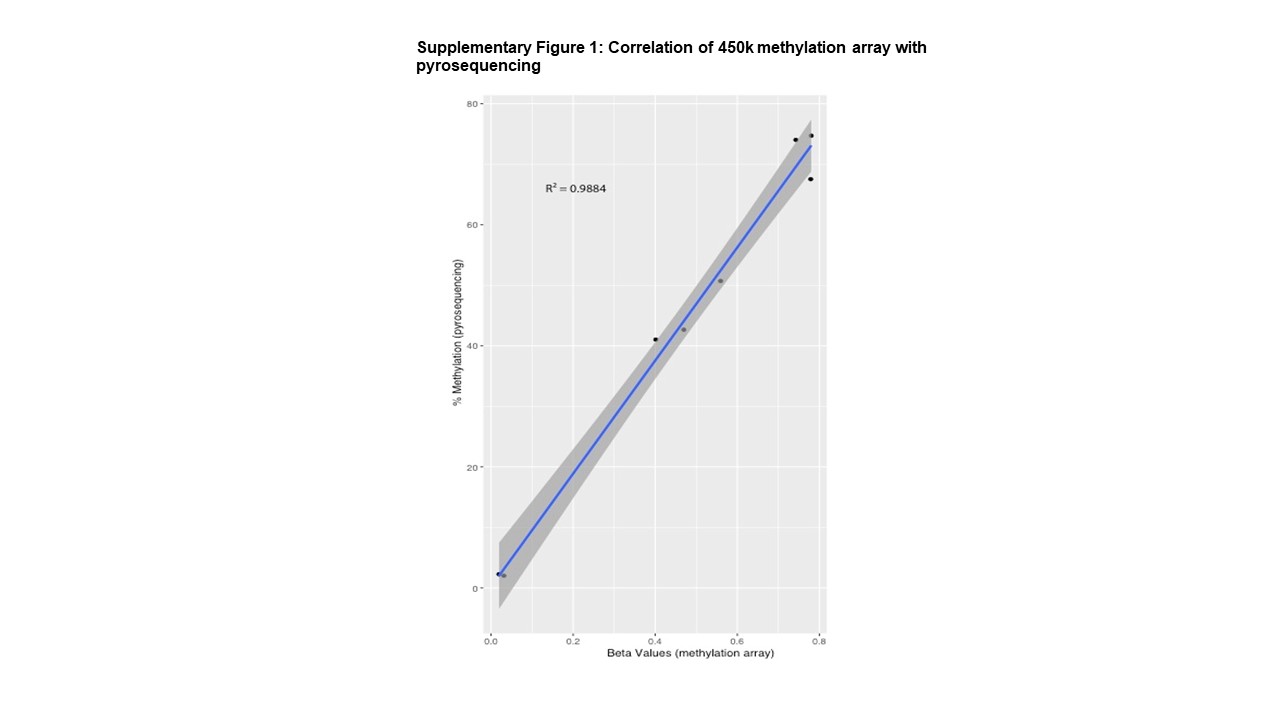

Supplement: Supplementary file 2 [file Image1.JPEG]
